# A Location-allocation Model for Bio-waste Management in the Hospitality Sector

**DOI:** 10.1007/s11067-023-09593-0

**Published:** 2023-06-10

**Authors:** Dolores R. Santos-Peñate, Rafael R. Suárez-Vega, Carmen Florido de la Nuez

**Affiliations:** 1 Dpto de Métodos Cuantitativos en Economía y Gestión/TIDES, Universidad de Las Palmas de Gran Canaria, Municipality of Las Palmas de G.C., Spain; 2 Dpto de Análisis Económico Aplicado/TIDES, Universidad de Las Palmas de Gran Canaria, Municipality of Las Palmas de G.C., Spain

**Keywords:** Circular economy, Tourism, Sustainability, Mixed integer programming model

## Abstract

Tourism generates huge amounts of waste. It has been estimated that about half of the waste generated by hotels is food and garden bio-waste. This bio-waste can be used to make compost and pellets. In turn, pellets can be used as an absorbent material in composters and as an energy source. In this paper, we consider the problem of locating composting and pellet-making facilities so that the bio-waste generated by a chain of hotels can be managed at or close to the generation points. The general objective is twofold: i) to avoid waste transportation from generation to treatment points and product transportation from production to demand points, and ii) to implement a circular model in which the hotels themselves become the suppliers of the products they need (compost and pellets) by transforming the bio-waste that they generate. Any bio-waste not processed by the hotels has to be treated at private or state-run plants. A mathematical optimization model is presented to locate the facilities and allocate the waste and products. The application of the proposed location-allocation model is illustrated with an example.

## Introduction

Tourism is an economic activity that involves the movement of individuals to places other than their residences for entertainment or leisure purposes. Based on data provided by destinations, the United Nations World Trade Organisation reported a significant growth in tourist arrivals (overnight visitors) of 4% in 2019, although this was slightly lower than the growth rate of 2017 and 2018. Before the Covid-19 crisis, a further worldwide rise of 3% to 4% had been estimated for 2020 (UNWTO 2020). In Spain, the contribution of tourism to the economy in 2018 was 12.3% of GDP (Instituto Nacional de Estadística (INE) 2019). This contribution varies between the country’s different regions, rising to as high as 35% of GDP in the Canary Islands as well as providing 40.40% of the employment generated in the islands in 2018 (EXCELTUR 2018).

While tourism clearly plays an important role in the economy, it also has a negative impact from the point of view of resource consumption and waste generation. These drawbacks are commonly aggravated by the concentration of visitors in time and space, and by the fact that some destinations may not be designed to withstand the pressure that comes from inadequate waste management (incorrect separation of different types of waste, failure to control its removal through integrated systems, shortage of skilled and qualified waste managers, etc). In the hotel sector in particular, the huge pressure on resources (energy, water, land and materials such as fossil fuels, minerals, metals and biomass) can have significant negative external impacts (congestion problems, loss of biodiversity, $CO_2$ emissions and environmental contamination). Circular economy models have been proposed as an alternative to the way resources and waste are managed, recycled and reused in the tourism sector and, in particular, in the hotel industry (Florido et al. 2019; Ghisellini et al. 2015; He et al. 2018; Rodríguez et al. 2020). In a more general framework, the concept of circular economy has been incorporated in theoretical models of economic growth, giving rise different conclusions depending on the circularity introduced in the economic system (Donaghy 2022).

The results of studies about the impact of tourism on waste generation have not always been in agreement. While some papers have concluded that a tourist generates about twice as much waste as a resident (Shamshiry et al. 2011), others have reported that the contribution per tourist to waste generation through the main tourism activities is less than that produced per resident in the residential and economic sectors (Díaz-Farina et al. 2020). These differences in estimations could be due to the properties of the particular scenarios studied or/and to the methodologies used in the respective analyses. Among the different types of waste generated by the hospitality sector, it should be noted that a large proportion is food waste, mainly from restaurants and kitchens. Some studies found that around 40% of all the waste generated in the hospitality sector is food waste (Castiglioni Guidoni et al. 2018; Pirani and Arafat 2014), while another reported that 47% of hospitality waste sent to landfill in the UK was food (Williams et al. 2011). In the paper by Papargyropoulou et al. (2016), after analysing the generation of food waste in a restaurant located in a 5-star hotel in Kuala Lumpur, Malaysia, an average 1.1 kg of food waste per guest per day was estimated. In the study presented by Phu et al. (2018) of 120 hotels in a tourist city of Vietnam, it was found that the mean amount of waste generated was 2.28 kg/guest/day, with the amount for a particular hotel influenced by capacity, price, garden surface, type of restaurant, and managerial practices, among others. For this case study, the authors found that the highest amounts of waste corresponded to kitchen (35.5%) and garden (15.5%). These results give an idea of the magnitude of the food waste generated by the hospitality and tourism sector and suggest the urgent need to carry out actions to reduce the ecological footprint that it leaves behind. For further information, interesting reviews of food waste management in the hospitality sector can be found in Filimonau and De Coteau (2019) and Pirani and Arafat (2014).

Mitigation of the impact of food waste ranges from prevention strategies to recovery and recycling plans. In this paper, we are interested in bio-waste generated in hotels, which includes biodegradable waste from gardens and food waste from kitchens and restaurants, and the focus of our study is on the recycling stage. Biodegradable waste can be decomposed by the natural action of living organisms and recycled as compost, which can then be used to fertilize gardens, parks, agricultural fields, etc. Food waste can also be used to produce energy, animal feed, and other materials (Paritosh et al. 2017; Pfaltzgraff et al. 2013; Pöyry 2019). Plant remains from pruning or gardens can be used to make splinters and pellets. Splinters are small pieces of woods broken off from a larger piece, it can be produced from pallets used to transport goods and other wood waste. Wood pellets are a woody, cylindrical bio-fuel of varying length, typically from 3.15 to 40 mm, a diameter of about 6-8 mm, and with broken ends (ENplus standards) (European Pellet Council 2015). It is a high calorific value fuel (more than 4.6 kWh/kg) with zero $CO_2$ emissions (the emission in combustion is equal to the amount set by the plant during its growth). Splinters and pellets can be used both in the composting process and as bio-fuel. In hotel resorts, bio-fuel boilers can be used for pool heating, hot water supplies and heating systems (García Machín 2014). They can serve as a means of improving energy efficiency while giving the hotel brand a more attractive image from an environmental sustainability point of view. In the case of Gran Canaria (Canary Islands, Spain), the reference scenario for our study, pellets have to be shipped in from continental Europe due to the lack of local suppliers, which can mean they are often exposed to unfavourable humidity conditions. Of course, this also entails an additional transportation cost in the supply to hotels on the island when compared to those located in mainland Spain (Díaz Martín et al. 2015a, b). Although splinters are produced on the island, it is done on a very small scale. In general, the dependency on external markets and the costs associated with transportation do not favor the demand increase for these recycled materials. In Gran Canaria, most of the material resulting from hotel garden pruning is sent to waste processing installations run by the island’s Regional Government, where it is used to produce organic compost.

Situated off the northwest coast of Africa and over 1,500 km from mainland Spain, the continued energy dependency on imported fossil fuel resources and the scarcity of freshwater resources, makes the Canary Islands a highly vulnerable tourist destination. The most serious problems associated with the development of tourism in the islands are the consumption of the scant resources available and the generation of waste. The appropriate and sustainable management of resources is and should continue to be a key aspect of current and future tourism policies. If, as some studies have argued, a tourist generates around twice as much waste as a resident, and we consider a resident population in the islands of 2,153,389 inhabitants (year 2019) and an entry of 15,110,866 tourists with an average stay of 9.09 days, then tourists are responsible for 25.89% of the waste that is generated. For the particular case of Gran Canaria, with a resident population of 851,231 inhabitants (2019) and 3,732,083 tourist visitors staying on average 6.93 days (Instituto Canario de Estadística (ISTAC) 2019), tourists are responsible for 10.88% of the waste generated.

With the aim of reducing the unwanted effects of tourism on the environment, there is a growing interest in the Canary Islands in promoting sustainable tourism, understood as "tourism development that is ecologically sustainable in the long term, economically viable, as well as ethically and socially equitable" (BRESCE 2009). In line with the agreements of the World Charter For Sustainable Tourism +20 and the Sustainable Development Goals (SDGs), the island’s Regional Government has recently set up the Gran Canaria Sustainable Tourism project. The goal of this developmental project, whose legal basis is the Gran Canaria Special Territorial Plan for Waste (Spanish initials: PTER 2014/10/03), is to ensure an efficient waste management that is compatible with the environment and does not impact the island’s natural values. In accordance with the Plan, hotel resorts are considered large waste generators and are legally obliged to have an adequate waste management system. In order to facilitate and guide hotels on waste management, the Gran Canaria Regional Government has published a handbook on waste management in tourist establishments (Cabildo Insular de Gran Canaria 2017). Among other actions, hotels are encouraged to compost vegetable and food waste.

Many of the hotels in the Canary Islands have carried out actions that contribute to a more sustainable tourism. By way of example, the hotel chain Lopesan Hotel Group (2020) through the use of clean energies, the recycling of $60\%$ of the waste generated, and a rationalized use of its resources, has reduced its $CO_2$ emissions by 4500 MT. Also, the chain Be Cordial Hotels and Resorts (2020) has implemented bio-fuel (pellet) boilers to heat the water for sanitary consumption and its swimming pools. This innovation has produced a significant reduction in both pollution levels and costs (García Machín 2014). Some hotels use their bio-waste to produce compost. In Hotel La Palma & Teneguía Princess (Princess Hotels 2020), the bio-waste is separated at the hotel and transported to the *ecopark* treatment plant where it is transformed into compost which is then returned to the hotel. Another example is Tigaiga Hotel (2020) which generates its own compost from its waste and uses it in its gardens.

In this paper, we consider a location-allocation problem connected to the management of bio-waste generated in hotels. Our contribution is an optimization model to determine where to locate bio-waste processing facilities and how to allocate the waste generated to the treatment facilities, and the products made in them to demand points, in order to meet a set of objectives. Location models have been used in many contexts to determine the best locations for the development of specific activities (Drezner et al. (2020), Erkut and Neuman (1989), Karsu et al. (2021), Kaya and Ozkok (2020), Kuby and Church (2010), Marianov et al. (2020), Miralinaghi et al. (2017), Moura et al. (2012), Palacio et al. (2016), Shishebori et al. (2014), Zheng et al. (2017), Zhi and Keskin (2018), among others). In particular, these models have been applied to select sites for the installation of waste processing plants and landfills. Some examples can be found in Berglund and Kwon (2014), Eiselt and Marianov (2014), Eiselt and Marianov (2015), Ghiani et al. (2014), Hrabec et al. (2017), Karagiannidis et al. (2004), Yadav et al. (2017) and Yadav et al. (2018). These papers consider economic and environmental objectives and are focused on public facilities. Most of these models consider known waste generation points and a set of potential locations to install landfills and/or transfer stations, with a certain distance between them. Moreover, these models can incorporate different types of cost (installation, transportation and processing, among others), capacity and demand constraints, pollution considerations, legal regulations, processing constraints (input–output relation), and uncertainty in the values of the model parameters. Our problem has common elements with these location models but is somewhat different. We consider both public and private bio-waste facilities for the treatment of food and garden waste, and the allocation of different products (compost and pellets) of the recycling process to demand points. More specifically, we consider a hotel chain which plans to manage the bio-waste generated in its installations using their own treatment equipment. This means that a hotel can simultaneously develop the three roles, waste generator, processing center, and point of demand for the resulting product. In our problem, the installation of a new facility for the treatment of one type of waste may imply an increase in the demand for a product that can be obtained from the processing of another type of waste. The input–output constraints and demand constraints are formulated to incorporate the circular system in which product obtained by processing waste in a facility can be used as input in other waste treatment installation. The waste not processed in these facilities has to be transported to the public and private bio-waste facilities. As with other location problems, our model has the difficulty of including integer variables. Moreover, depending on the variable operating costs of the facilities, the model could be non-linear. The non-linearity can be solved by introducing some new variables and constraints, converting the problem into a linear model at the cost of increasing its size. On the other hand, similarly to other location problems, part of the model constraints have an advantageous formulation (variables with zero–one coefficients), and for problems that are not too large, a solution can be found in a reasonable time.

This paper is organized as follows. In Section 2 we state the problem. The location-allocation model is described in Section 3. In Section 4 the model size is calculated and some extensions of the problem are presented. Sections 5 and 6 show the application of the model to a specific scenario in Gran Canaria. Finally, Section 7 reports the conclusions of the study.

## Scenario and Problem Statement

Before stating the problem, we describe briefly the situation with respect to tourism in Gran Canaria which has motivated this work. The contribution of tourism to the economy of the island is very important and generates a significant amount of employment. Presently, the island can accommodate more than 152,000 tourist visitors at any one time, 44,138 of whom can opt to stay in 4 and 5 star hotels (Cabildo Insular de Gran Canaria 2020).

The south of the island is where most of the hotels are concentrated and the vast majority of tourist visitors stay. In 2019, $37\%$ of visitors stayed at 4-5 star hotels, and more than $50\%$ chose all-inclusive, half-board or bed and breakfast packages. It can be assumed that kitchens and restaurants in hotels, particularly in 4-5 star hotels, generate large amounts of food waste. Normally these hotels also have garden zones which generate garbage. In the largest hotels of Gran Canaria, food and garden waste are transported to the installations run by the island authorities. Here, part of the garden waste is used to produce compost, but most of the food waste goes to landfill.

In the framework of a sustainable economy, transportation should be avoided and food waste processed in the same hotels where it is generated or as close to them as possible. With this in mind, in the present study, we consider a hotel chain which plans to install facilities (composters and pelletizers) to process the food and garden waste generated in their hotels. Given a set of candidate sites, the problem is to determine the location and type of the facilities to be installed so that the total cost is minimized. We assume that all the food and garden waste can be used to produce compost and pellets. The food waste and *garden-soft* waste (for example tree leaves) can be used to produce compost. The *garden-hard* waste (tree trunks and branches) can be used to make pellets or splinters. In turn, the compost can be used in the hotel gardens and farms belonging to the hotel chain or to partners in the agriculture sector, and pellets and splinters as absorbent material for composting and as a renewable energy source. Boilers working with bio-fuel are used to heat water to be consumed in the hotel. We take into account that a boiler of splinters admits pellets, but splinters can not always be used in pellet boilers. Despite these differences, and although pellets and splinters have different energy values and prices, for simplicity we consider both products as one which we call *pellets*.

Hotels generate bio-waste which can be transformed into products (compost and pellets) in composters and pelletizers. The products can then be distributed from the suppliers to the demand points, including the hotels and others. Since all waste has to be processed in some way, waste that is not treated in hotels or private facilities has to be taken to the ecopark. Ecoparks are enclosed controlled areas with waste treatment equipment and landfill which, in Gran Canaria, are managed by the *Cabildo Insular*. It is assumed that pellets have to be imported if demand cannot be satisfied by the hotels’ own production and the small amount of pellets available from local suppliers. It is also assumed in the model that hotels may be owners of agricultural areas, which are waste generators and can be considered candidate locations for the instalment of bio-waste treatment facilities or/and product distribution points.

## The Model

In this section, we present a location-allocation model to determine the number of facilities to be installed, where to locate the facilities, and how to plan production and allocate the products (compost and pellets) to satisfy the demand, in order to minimize the total cost. We consider three types of waste (food, garden-soft, and garden-hard) and two products (compost and pellets). There are two categories of facility, composters (category 1) and pelletizers (category 2). For each category, we have several types or models characterized by properties such as size, capacity, energy consumption, etc. The decision maker (hotel chain) wants to determine how many facilities to install, where to install the facilities, the type of facility to be installed, the waste to be treated at each facility, and the distribution of the products among the demand points, in order to minimize the total cost. We assume that there are no waste treatment facilities at the hotels belonging to the chain. Moreover, we consider that, for private treatment plants not belonging to the hotel chain, a limitation on the amount of waste admitted for treatment may exist. This limitation and the unit price can be established through commercial agreements. With respect to the ecopark, the capacity is assumed to be infinite so that the waste not processed in other places can go there.

We now introduce the model.

### Sets

$$I= \{ i\}_{i=1}^{i=n_I} = \{1,...,n_I\}$$
index set for nodes (waste generation points, treatment points, demand points, suppliers). Here $i=n_I$ corresponds to the ecopark.
$$F= \{ f\}_{f=1}^{i=2} = \{1,2\}$$
index set for categories of facility. Here $f=1$ and $f=2$ indicate composter and pelletizer, respectively.
$$K_f= \{ k\}_{k=1}^{k=n_{K_f}} = \{1,...,n_{K_f}\}$$
index set for types of facility of category $f \in F$. For each category (composter and pelletizer), we can have different types determined by the capacity or other properties of the machine.
$$J \subseteq I$$
index set for candidate locations to install facilities. Moreover, *J* is the set of waste generators and the set of demand points. Set *J* represents the set of hotels belonging to the chain.
$$U \subseteq I$$
index set for locations where facilities already exist.

We have $$J \cap U = \emptyset$$.

$$A= \{ a\}_{a=1}^{a=3} = \{1,2,3\}$$
index set for types of bio-waste. Here $a=1$ indicates food waste, $a=2$ garden-soft waste and $a=3$ garden-hard waste.
$$B= \{ b\}_{b=1}^{b=2} = \{1,2\}$$
index set for types of product. Here $b=1$ indicates compost and $b=2$ pellets.
$$S_b \subset I$$
index set for suppliers of product *b* other than the hotels in the chain.
$$\hat{AB}= \{(1,1),(2,1),(3,2)\}$$
subset of $A \times B$ consisting of pairs (*a*, *b*) such that waste type *a* can be transformed into product *b*.

### Parameters

$$c^C_{jkf}$$
capital cost for facility of category $f \in F$ and type $k \in K_f$ at node $j \in J$ per year.
$$c^{FX}_{jkf}$$
fixed cost for facility of category $f \in F$ and type $k \in K_f$ at node $j \in J$ per year.
$$c^V_{jkf}$$
variable cost of facility of category $f \in F$ and type $k \in K_f$ at node $j \in J$ per unit of material processed.
$$c^T_{aji}$$
transportation cost from node $j \in J$ to node $i \in J \cup U$ per unit of waste type $a \in A$.
$$\hat{c}^T_{bij}$$
transportation cost from node $i \in J \cup S_b$ to node $j \in J$ per unit of product type $b \in B$.
$$c^S_b$$
unit surplus cost for product $b \in B$.
$$\pi _{abu}$$
unit price of treatment of waste $a \in A$ to obtain product $b \in B$ at node $u \in U$, for $(a,b) \in \hat{AB}$.
$$\hat{\pi }_{bs}$$
unit price of product $b \in B$ at node $s \in S_b$.
$$g_{aj}$$
amount of waste of type $a \in A$ generated at node $j \in J$ per year.
$$e_{kf}$$
capacity of facility of category $f \in F$ and type $k \in K_f$ per year.
$$\hat{e}_{u}$$
waste reception capacity for existing treatment plant located at node $u \in U$ per year (limitation on the amount of waste admitted for treatment).
$$q_{bj}$$
demand of product $b \in B$ at node $j \in J$ per year, excluding the amount of pellets required in the production of compost, which is determined by the model.
$$\rho _{b}$$
production coefficient, amount of product of type $b \in B$ per unit of material processed in a facility (product = $\rho \times$ material). We assume that the production coefficient does not depend on the type ($k \in K_f$) of the facility category ($f \in F$).
$$\tau$$
amount of pellets (absorbent material) per unit of waste required to produce compost in a composter.
$$\tilde{e}_{bs}$$
capacity of supplier $s \in S_b$ to provide product $b \in B$.

### Variables

$$y_{jkf}$$
binary variable, $y_{jkf}=1$ if a facility of category $f \in F$ and type $k \in K_f$ is installed at node $j \in J$, otherwise $y_{jkf}=0$.
$$x_{abji}$$
amount of waste of type $a \in A$ from node $j \in J$ transformed into product $b \in B$ at node $i \in J \cup U$, for $(a,b) \in \hat{AB}$.
$$\hat{x}_{bij}$$
amount of product of type $b \in B$ from node $i \in J \cup S_b$ assigned to demand node $j \in J$.

It could occur that, due to installation requirements, some specific facility types cannot be sited in certain candidate locations, for example when the size of a particular type of composter is too large and requires a larger space than is available in the hotel. To incorporate these limitations in the formulation we would consider the set of candidate locations for each category and type, and we would define the variables $y_{jkf}$ accordingly.

### Objective Function

The objective function is the total cost resulting from the summation of the capital cost, operating cost (fixed cost plus variable cost), transportation cost, treatment cost, product cost, and surplus cost. The total cost is denoted as *TOTCOST*, and is defined as

1 $$\begin{aligned} TOTCOST = CC + OC+ TC+ TreC+PC+SC. \end{aligned}$$

where *CC*, *OC*, and *TC* denote capital cost, operating cost, and transportation cost, respectively. *TreC* is the treatment cost, *PC* is the product cost, and *SC* is the surplus cost. That is:

2 $$\begin{aligned} CC = \sum \limits _{j \in J,k \in K_f, f\in F} c^C_{jkf} y_{jkf} \end{aligned}$$

3 $$\begin{aligned} \begin{array}{ll} OC = &{} \sum \limits _{j \in J,k \in K_f, f\in F} c^{FX}_{jkf} y_{jkf} + \\ &{} \sum \limits _{i \in J,j \in J, k\in K_1} c^V_{jk1} (1+\tau )(x_{11ij}+x_{21ij})y_{jk1} + \sum \limits _{i \in J,j \in J, k\in K_2} c^V_{jk2}x_{32ij}y_{jk2}. \end{array} \end{aligned}$$

4 $$\begin{aligned} TC = \sum \limits _{(a,b) \in \hat{AB},j \in J,i \in J \cup U} c^T_{aji} x_{abji} + \sum \limits _{b \in B,i \in J \cup S_b,j\in J} \hat{c}^T_{bij} \hat{x}_{bij}. \end{aligned}$$

5 $$\begin{aligned} TreC = \sum \limits _{(a,b) \in \hat{AB},j \in J, u \in U} \pi _{abu} x_{abju}. \end{aligned}$$

6 $$\begin{aligned} PC = \sum \limits _{b \in B,s \in S_b,j \in J} \hat{\pi }_{bs} \hat{x}_{bsj}. \end{aligned}$$

7 $$\begin{aligned} SC = \sum _{b \in B, i \in J \cup S_b, j \in J } c^S_b ( \hat{x}_{bij} - (q_{bj}+ \Phi _{bj})) \end{aligned}$$

where $q_{bj}$ is the demand already defined and $\Phi _{bj}$ is a function of variables $x_{abji}$. Here, $\Phi _{1j}=0$ and $\Phi _{2j} = \tau \sum \limits _{a=1,2, i \in J } x_{a1ij}$. Observe that, for pellets, the total demand includes the amount required for composters and demand for other uses. If $q_{bj}$ is the pellet demand for other uses, total pellet demand at node $j \in J$ is $q_{bj} + \tau \sum \limits _{a=1,2, i \in J} x_{a1ij}$.

The surplus cost (SC) is associated to the surplus of compost or pellets. In the case of surplus cost (storage cost, transfer to deposit or ecopark), the corresponding values are incorporated in the objective function. On the other hand, in some cases, the price fixed by the service or product supplier includes the transportation cost.

### Constraints

For each candidate location, at most one facility of category *f* is installed:

8 $$\begin{aligned} \sum \limits _{k \in K_f}y_{jkf} \le 1 \qquad \forall j \in J, \; f \in F. \end{aligned}$$

Equipment capacity constraints (composters and pelletizers) for candidate locations:

9 $$\begin{aligned} (1+\tau ) \sum \limits _{a=1,2, i\in J } x_{a1ij} \le \sum \limits _{k \in K_1}e_{k1}y_{jk1} \qquad \forall j \in J. \end{aligned}$$

10 $$\begin{aligned} \sum \limits _{i\in J}x_{32ij} \le \sum \limits _{k \in K_2}e_{k2}y_{jk2} \qquad \forall j \in J. \end{aligned}$$

Constraints (9) say that the amount of material deposited in a composter (facility of category 1) installed at location $j \in J$, cannot be greater than the capacity of the machine to be installed. Taking into account that $\tau$ is the amount of pellets (absorbent material) required per unit of waste, the amount of material to be introduced in the composter is given by the expression on the left side of the constraints. Constraints (10) are the capacity constraints for pelletizers. On the other hand, constraints (9) and (10) force waste not to be assigned to node $j \in J$ if a treatment facility is not installed there.

Treatment capacity constraints for existing facilities:

11 $$\begin{aligned} \sum \limits _{(a,b) \in \hat{AB}, j\in J} x_{abju} \le \hat{e}_{u} \qquad \forall u \in U \setminus \{n_I\}. \end{aligned}$$

Supply capacity constraints:

12 $$\begin{aligned} \sum \limits _{j\in J} \hat{x}_{bsj} \le \tilde{e}_{bs} \qquad \forall s \in S_b, b \in B. \end{aligned}$$

All waste is treated:

13 $$\begin{aligned} \sum \limits _{b\in B, \text { with } (a,b) \in \hat{AB},i \in J \cup U }x_{abji} = g_{aj} \qquad \forall a\in A,\; j \in J. \end{aligned}$$

Demand constraints:

14 $$\begin{aligned} \sum \limits _{i \in J \cup S_b}\hat{x}_{bij} \ge q_{bj}+ \Phi _{bj} \qquad \forall b\in B, j \in J, \end{aligned}$$

where $$\Phi _{1j}=0$$ and $$\Phi _{2j} = \tau \sum \limits _{a=1,2, i \in J } x_{a1ij}$$.

Production or input–output constraints for compost:

15 $$\begin{aligned} \sum \limits _{j \in J}\hat{x}_{1ij} = \rho _{1}(1+\tau ) \sum \limits _{a=1,2, j \in J} x_{a1ji} \qquad \forall i \in J. \end{aligned}$$

Production or input–output constraints for pellets:

16 $$\begin{aligned} \sum \limits _{j \in J}\hat{x}_{2ij} = \rho _{2}\sum \limits _{j \in J}x_{32ji} \qquad \forall i \in J. \end{aligned}$$

In constraints (15) and (16), the left hand side is the amount of product (compost and pellets, respectively) obtained at node *i*, the right hand side is the amount of material introduced into the machine (composter and pelletizer) multiplied by the production coefficient.

Domain constraints:

17 $$\begin{aligned} \begin{array}{l} y_{jkf} \in \{0,1\}, \; \forall j \in J, k \in K_f, f \in F \\ x_{abji} \ge 0, \; \forall (a,b) \in \hat{AB}, j \in J, i \in J \cup U \\ \hat{x}_{bij} \ge 0, \forall b \in B, i \in J \cup S_b, j \in J. \end{array} \end{aligned}$$

The optimization problem we have to solve is:

18 $$\begin{aligned} \min TOTCOST \text { subject to (8)-(17) } \end{aligned}$$

where *TOTCOST* is defined by Eqs. (1)-(7).

Note that Problem (18) does not include budget constraints. In some situations, the chain could have budget restrictions which can be expressed as a limitation on the total cost or the number of facilities to be installed. In the first case, if $\Gamma$ represents the budget, the constraint $TOTCOST \le \Gamma$ would be added. In the second case, a limitation on the number of facilities would be incorporated into the model. For each category *f*, the number of facilities (formulated as $\sum \limits _{j\in J, k \in K_f}y_{jkf}$) would have to be less than or equal to a given value.

It can be observed that all the constraints of the model are linear. If the variable cost depends on the type of facility (*k*), the operation cost (*OC*) is a non-linear function giving a mixed integer non-linear problem. In that case, we can linearize this optimization problem by introduction of the variables $z_{jkf}$, with $j \in J, k \in K_f, f\in F$, defined as follows:

19 $$\begin{aligned} z_{jkf} = \left\{ \begin{array}{lcl} (1+\tau )\sum \limits _{a=1,2, i\in J } x_{a1ij} &{} if &{} f=1 \text { and } y_{jk1}=1\\ \sum \limits _{i\in J }x_{32ij} &{} if &{} f=2 \text { and } y_{jk2}=1 \\ 0 &{} if &{} y_{jk1}=y_{jk2}=0.\\ \end{array}\right. \end{aligned}$$

Then, using these variables, the variable cost is $\sum \limits _{j \in J, k \in K_f, f\in F} c^V_{jkf}z_{jkf}$. The definition of the variables $z_{jkf}$ is introduced in the formulation by adding the following constraints:

20 $$\begin{aligned} \left\{ \begin{array}{lcl} z_{jk1} - (1+\tau )\sum \limits _{a=1,2, i\in J }x_{a1ij} &{} \ge &{} -M(1-y_{jk1}) \\ z_{jk2} - \sum \limits _{i\in J }x_{32ij} &{} \ge &{} -M(1-y_{jk2}) \\ \end{array}\right. \end{aligned}$$

where *M* is a big enough number.

This linearization procedure is not necessary if $c^V_{jkf}=c^V_{jf}$, that is, if the cost does not depend on the type (*k*) of facility. In that case:

$$OC = \sum \limits _{j \in J,k \in K_f, f\in F} c^{FX}_{jkf} y_{jkf} + (1+\tau ) \sum \limits _{a=1,2, i,j \in J} c^V_{j1} x_{a1ij} + \sum \limits _{i,j \in J} c^V_{j2}x_{32ij}.$$

## Model Size and Model Generalization

For any set *S*, we use the notation |*S*| to represent the cardinality of *S*. Then, the model given by (18) has the number of variables and constraints indicated in Table 1 (excluding domain constraints).

**Table 1:** Model size

Number of variables	
$y_{jkf}$ (binary)	$|J| \times \sum \limits _{f \in F} |K_f|$
$x_{abji}$ (continuous)	$|\hat{AB}|\times |J| \times |J \cup U|$
$\hat{x}_{bij}$ (continuous)	$|J| \times \sum \limits _{b \in B} |J \cup S_b|$

Number of constraints	
(8)	$$|F|\times |J| = 2 \times |J|$$
(9), (10)	$$|F|\times |J| = 2 \times |J|$$
(11)	$$|U|-1$$
(12)	$$\sum \limits _{b \in B} |S_b|$$
(13)	$$|A|\times |J| = 3 \times |J|$$
(14 )	$$|B| \times |J| = 2 \times |J|$$
(15), (16)	$$|F| \times |J| = 2 \times |J|$$
Total constraints	$11\times |J| + \Big ( |U|-1\Big ) + \sum \limits _{b \in B} |S_b|$

The model can be adapted to general situations where the planner considers the location of $n_F$ facility categories for treatment of $n_A$ types of bio-waste providing $n_B$ types of products. Moreover, the problem could be extended to the case in which a type of waste is used to obtain several products. An example is the use of food waste for providing compost and biogas. Other extension is to consider different facility categories giving the same product with different technologies. We can also consider the case in which obtaining a product from waste treatment requires adding more than one product to the waste. As an example, suppose that category *f* generates product *b* by processing of waste *a*, with $(a,b) \in \hat{AB}$, using products in $B_b^{in} \subset B$, with $\tau _{lb}$ the amount of product $l \in B_b^{in}$ required per unit of waste. Consider that product *b* can be used to obtain other products. In this case the model would be modified as follows. The variable cost in the objective function would have to be reformulated. Constraints (8), (11), (12), (13), and (17) remain the same. The equipment capacity constraints corresponding to product *b* and category *f* would be

21 $$\begin{aligned} \sum \limits _{a / (a,b) \in \hat{AB}, i \in J} \Big ( 1+ \sum \limits _{l \in B_b^{in}} \tau _{lb}\Big ) x_{abij} \le \sum \limits _{k \in K_f}e_{kf}y_{jkf} \qquad \forall j \in J, \end{aligned}$$

which say that the amount of material introduced into facility of category *f* generating product *b* and located at point *j* cannot exceed its capacity.

The demand constraint for product $b \in B_l^{in}$ to produce *l* at $j \in J$ would be formulated as follows,

22 $$\begin{aligned} \sum \limits _{i \in J \cup S_b}\hat{x}_{bij} \ge q_{bj}+ \Phi _{bj} \end{aligned}$$

where

23 $$\begin{aligned} \Phi _{bj} = \sum \limits _{a / (a,l) \in \hat{AB},i \in J} \tau _{bl}x_{alij}. \end{aligned}$$

The input–output constraint for node $i \in J$ where product $b \in B$ is produced using products in $B_b^{in}$ would be the following

24 $$\begin{aligned} \sum \limits _{j \in J}\hat{x}_{bij} = \rho _{b}(1+\sum \limits _{l \in B_b^{in}}\tau _{lb}) \sum \limits _{a/ (a,b) \in \hat{AB}, j \in J}x_{abji}. \end{aligned}$$

## Illustrative Example

In order to illustrate the application of the model, we consider a set of 11 hotels belonging to the same firm and located on the island of Gran Canaria. This hotel chain, which actually operates in the south of the island, has a farm (agriculture area) that provides some products for consumption in the hotels. We assume that the bio-waste generated in the farm is managed *in situ*, transforming it into compost and pellets. Moreover, waste may be transported to the farm to be processed, it is assumed that the cost of waste treatment on the farm is zero. The study includes the closest ecopark, which is located in the same municipality where the hotels are situated. The ecopark supplies compost but not pellets. We suppose that a private treatment plant located in the area of interest is already operating and transforms bio-waste into compost and small amounts of pellets. For the farm and the local plant, the supply capacity for pellets is 10 tons per year. No waste from the hotels is processed in the non-local plant which acts solely as a supplier of pellets. Taking into account the accommodation capacity of the hotels, the amount of waste generated was calculated considering results found in the literature (Chan and Lam 2001; Papargyropoulou et al. 2016; Phu et al. 2018) and the occupancy ratio. The amount of food waste was estimated assuming 70% occupancy and 0.67 kg of waste generated per person and day. Although these hotels do not currently use pellet boilers, for illustrative purposes we assume that two of them, hotels 7 and 11, have this type of installation with a demand of 75 and 204 tons/year, respectively. The demand of compost was calculated taking into account the size of the hotel (number of beds). Travel times between nodes were calculated as the fastest route between them through the local road network. The calculations were done using the GIS ArcMap software.

In the tables and figures where the results are shown, labels H, FARM, LP, NLP and EC represent hotel, farm, local treatment plant, non-local treatment plant and ecopark, respectively. Labels FW and GW represent food waste and garden waste, respectively. Eight types of composter and one type of pelletizer are considered. The types and their capacities correspond to real commercialized products. Since the capacity of machine *f* of type *k* varies between values $min_{kf}$ and $max_{kf}$, we assumed that the capacity is equal to $min_{kf}+\alpha (max_{kf}-min_{kf})$, where $0< \alpha \le 1$ (composter capacity varies depending on several factors such as content/mix of food waste and how the machine is programmed). The values for the parameters $\tau$ and $\rho _b$, $b=1,2$, were chosen in the intervals [0.10, 0.20], [0.15, 0.25] and [0.9, 1], respectively. These intervals were selected based on the properties of existing treatment machines on the market. For the capital cost calculation, we present three scenarios for the discount rate, $\gamma =0, 0.05, 0.12$, and 25 years of technology life. We consider several scenarios for the treatment price in the ecopark (ECP), the unit transportation cost (UTC), and the unit surplus cost (USC). The parameter values used in the example, including the treatment and product prices, are summarized in Table 2. The total number of scenarios is $3 \times 5 \times 3 \times 4=180$.

For all scenarios, the model (18) has $11\times (8+1)=99$ binary variables ($y_{jkf}$), $3 \times 11 \times 14 + 11 \times 28 =462+308=770$ continuous variables ($x_{abji}$ and $\hat{x}_{bij}$), and 130 constraints (excluding the domain restrictions). For both categories (composter and pelletizer), we consider that the variable cost does not depend on the type, therefore the model is linear. The results (exact solutions) were obtained using a PC Intel(R) Core(TM) i7-6820HQ CPU 2.70GHz RAM 8 GB, with GAMS 39 and the solver CPLEX 22.1.0. For each discount rate scenario, the execution time consumed by GAMS to solve the 60 scenarios considered (including reading data and writing results) was less than 90 s. The average execution time to solve the problem for a scenario was 0.40 s.

**Table 2:** Parameter values

Parameter		Value	
$\alpha _1$, $\alpha _2$	machine capacity coefficient	0.5, 0.5	
$\tau$	absorbent material coefficient	0.15	
$\rho _1$, $\rho _2$	production coefficient	0.25, 0.99	
$\gamma$	discount rate (3 scenarios)	0, 0.05, 0.12	
$\lambda$	technology life	25 years	

Treatment prices (€/ton, any type of waste)			
LP		90**	
ECP	5 scenarios	90*, 100*, 120*, 150*, 200*	
Product prices		Compost	Pellet
LP		150*	200*
NLP		-	397.57*
EC		0**	-

Costs (€)			
UTC (€$/s \times ton$)	3 scenarios	0.016, 0.050, 0.150	
USC (€/*ton*)	4 scenarios	0, 10, 25, 50	

* including transport

** transport is not included

## Results

Tables 3, 4, and 5 show the results for the $5 (ECP)\times 3 (UTC) \times 2 (USC) = 30$ scenarios considering USC = 0 and USC = 25, with a discount rate equal to 0.12. Tables 3, 4 and 5 correspond to $UTC=0.016$, 0.050 and 0.150, respectively. For each table, rows 1 and 4, labelled as NC and NP, show the optimal number of composters (NC) and the optimal number of pelletizers (NP) for the scenarios indicated in columns. Rows 2 and 3 contain the nodes where a composter is installed and the type. The locations for pelletizers are indicated in row 5. Rows 6 to 13 show the amount of food waste (FW) and garden waste (GW) treated in the hotels (H), farm (FARM), local plant (LP), and ecopark (EC). Rows 14 to 17 show the compost provided by hotels (C-H), farm (C-FARM), local plant (C-LP) and ecopark (C-EC). Rows 18 to 21 show this information for pellets. Rows 22 and 23 show the surplus of compost (SC) and pellets (SP), respectively. Rows 24 to 30 show the total cost (TotC), the facility cost (FacC), which is the cost associated to the composters and pelletizers installed, the waste transportation cost (TraC-W), the product transportation cost (TraC-P), the treatment cost (TreC), the product cost (ProC), and the surplus cost (SurC). The treatment cost refers to the services in the ecopark and the local plant. Columns 1 and 2 contain the row numbering and its label respectively. Columns 3 to 7, and 8 to 12, show the results for scenario USC = 0 and USC = 25, respectively. So, for example, in Table 3, we see that for $UTC=0.016$ and $USC=0$, and for all ecopark price value, three composters are installed in the hotels, while for $USC=25$ no composter is installed. If the surplus cost is small enough, the hotel chain can obtain an economic benefit by installing composters because the reduction in weight produced by the composting process is significant. If the surplus cost increases, then the number of facilities decreases when the reduction in cost provided by the treatment of waste in the hotels does not compensate the surplus cost.

In Tables 3, 4, and 5, we observe that only for UTC = 0.016, some waste is treated in the farm. Since the farm is located some distance from the area where the hotels are located, it is used as a treatment point or product supplier only if the transportation cost is low enough. Moreover, for all scenarios only one pelletizer is installed. As expected, the number of composters tends to grow when the transportation cost and/or the treatment cost increases, and tends to decrease when the surplus cost increases. For the highest transportation cost (UTC = 0.15), the variability of the number of composters with respect to the treatment cost in the ecopark is more pronounced. It can be observed that for most scenarios the compost required by the hotels is provided by themselves and in all cases no pellet surplus exists. Last rows of the tables show that, for UTC = 0.016, the costs for all the ECP scenarios are the same, due to the coincidence in the facilities installed and the waste and product allocation. The case is different for UTC = 0.050 and UTC = 0.150.

Comparing scenarios UTC = 0.050 and UTC = 0.150, when USC = 25 and ECP = 100, we observe that an increase in transportation cost does not necessarily imply an increase in the number of facilities. For UTC = 0.050, four facilities are installed at nodes 1, 4, 9 and 10, and although node 3 is closer to node 1 than to node 4, due to the capacity limitations of the composter in hotel 1, the food waste from node 3 is assigned to node 4, which also receives the waste from nodes 4 and 5. For UTC = 0.150, three facilities are installed at nodes 1, 9 and 10, the food waste from nodes 1, 3 and 6 is treated at node 1 while that of nodes 2, 4 and 5 goes to the ecopark.

**Fig. 1:**
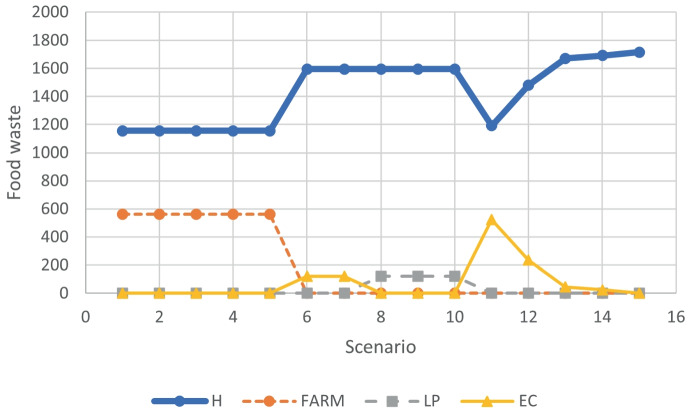
Food waste treated for scenarios with $USC=0$ and $\gamma =0.12$

**Fig. 2:**
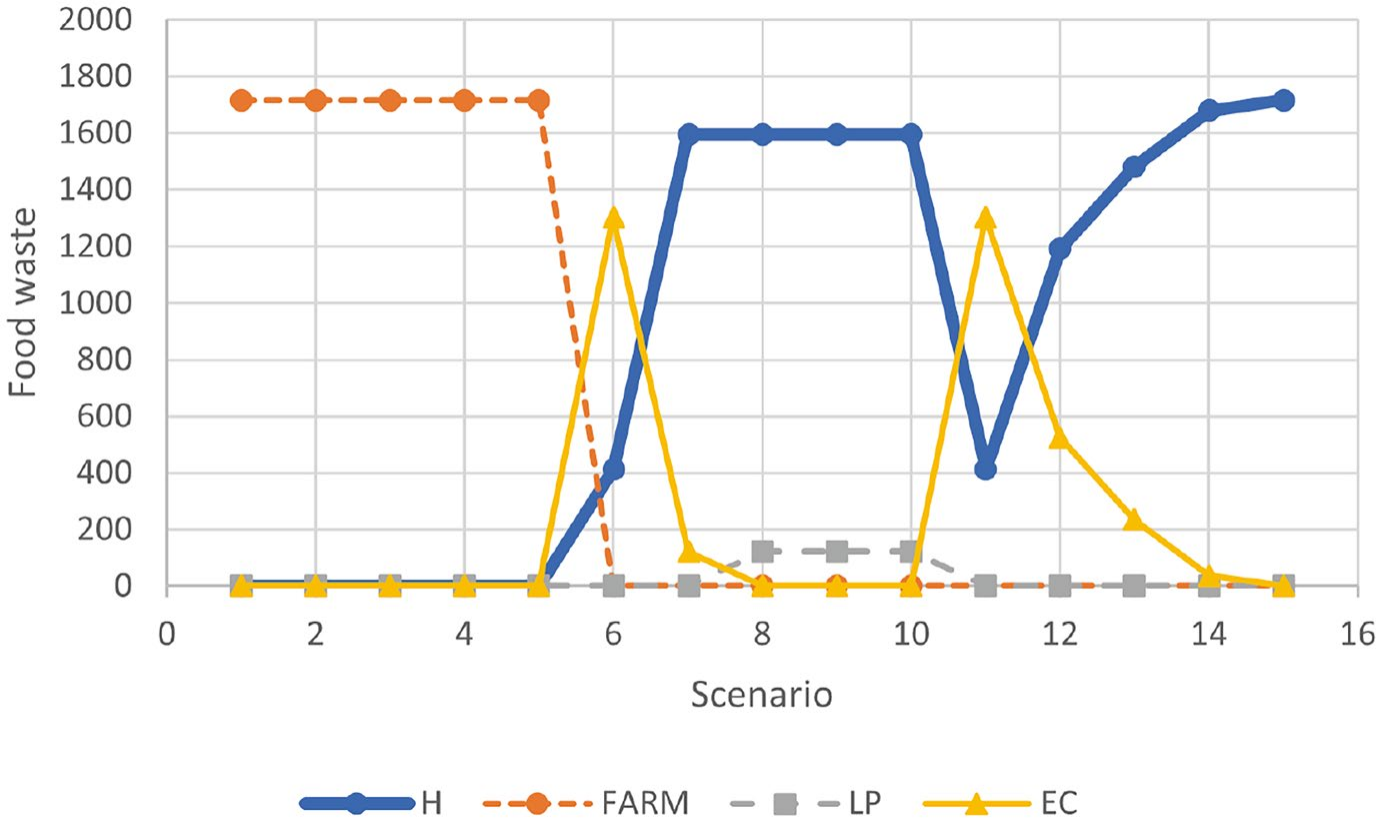
Food waste treated for scenarios with $USC=25$ and $\gamma =0.12$

Figures 1 and 2 summarize the food waste allocation information for the 15 (UTC,ECP) scenarios, for USC = 0 and USC = 25 respectively. Scenarios 1 to 5 correspond to UTC = 0.016, for the different ECP values. Analogously, for scenarios 6 to 10 and UTC = 0.050, and scenarios 11 to 15 and UTC = 0.150. Figure 1 (USC = 0) shows that for the lowest UTC value (UTC = 0.016), the amount of waste treated in the different types of establishment (hotel, farm, local plant and ecopark) is constant, the same for all scenarios (from 1 to 5), all food waste is processed in the hotels and the farm. For UTC = 0.050, the amount of waste treated in the hotels is constant, the variation of the ecopark price affects to the amount of waste allocated to the local plant (used for scenarios 8 to 10) and the ecopark (used for scenarios 6 and 7), no waste is treated in the farm. From scenarios 11 to 15, for which the UTC value is the highest, the amount of food waste treated in the hotel increases when the ECP rises. For these scenarios only the hotels and the ecopark receive waste from hotels to be processed.

Figure 2 shows that, for scenarios 1 to 5, due to the low transportation cost (UTC = 0.016) and the hight surplus cost (USC = 25), all the food waste is treated in the farm, where facilities are assumed to be already operating and only transportation cost is considered. As for USC = 0, for scenarios 6 to 10, part of the food waste goes to hotels and other part is processed in the ecopark (scenarios 6,7) and the local plant (scenarios 8,9 10). For scenarios 11 to 15, although with different amounts, the tends of the food waste allocation curves is similar to the one observed for USC = 0. For this illustrative example, the results show that, except for the case where USC = 25 and UTC = 0.016, the location of treatment facilities in some hotels is profitable for the chain.

**Table 3:** Results ($$\gamma =0.12$$)

**Scenario**		**UTC = 0.016** €$/ s \times ton$									
**USC** (€ /*ton*)		0					25				
**ECP** (€ /*ton*)		90	100	120	150	200	90	100	120	150	200
**Row**	**Label**										
1	NC	3	3	3	3	3	0	0	0	0	0
2	Nodes	1,4,10	1,4,10	1,4,10	1,4,10	1,4,10					
3	Type	8,8,8	8,8,8	8,8,8	8,8,8	8,8,8					
4	NP	1	1	1	1	1	1	1	1	1	1
5	Nodes	9	9	9	9	9	9	9	9	9	9
6	FW-H	1156.07	1156.07	1156.07	1156.07	1156.07	0.00	0.00	0.00	0.00	0.00
7	FW-FARM	560.58	560.58	560.58	560.58	560.58	1716.64	1716.64	1716.64	1716.64	1716.64
8	FW-LP	0	0	0	0	0	0	0	0	0	0
9	FW-EC	0	0	0	0	0	0	0	0	0	0
10	GW-H	578.18	578.18	578.18	578.18	578.18	73.82	73.82	73.82	73.82	73.82
11	GW-FARM	159.97	159.97	159.97	159.97	159.97	664.34	664.34	664.34	664.34	664.34
12	GW-LP	0	0	0	0	0	0	0	0	0	0
13	GW-EC	0	0	0	0	0	0	0	0	0	0
14	C-H	477.38	477.38	477.38	477.38	477.38	0	0	0	0	0
15	C-FARM	0	0	0	0	0	0	0	0	0	0
16	C-LP	0	0	0	0	0	0	0	0	0	0
17	C-EC	0	0	0	0	0	10.03	10.03	10.03	10.03	10.03
18	P-H	73.08	73.08	73.08	73.08	73.08	73.08	73.08	73.08	73.08	73.08
19	P-FARM	10.00	10.00	10.00	10.00	10.00	10.00	10.00	10.00	10.00	10.00
20	P-LP	10.00	10.00	10.00	10.00	10.00	10.00	10.00	10.00	10.00	10.00
21	P-NLP	434.99	434.99	434.99	434.99	434.99	185.92	185.92	185.92	185.92	185.92
22	SC	467.35	467.35	467.35	467.35	467.35	0	0	0	0	0
23	SP	0	0	0	0	0	0	0	0	0	0
24	TotC	282458.16	282458.16	282458.16	282458.16	282458.16	285362.98	285362.98	285362.98	285362.98	285362.98
25	FacC	45995.36	45995.36	45995.36	45995.36	45995.36	9411.57	9411.57	9411.57	9411.57	9411.57
26	TraC-W	25166.18	25166.18	25166.18	25166.18	25166.18	80544.12	80544.12	80544.12	80544.12	80544.12
27	TraC-P	331.09	331.09	331.09	331.09	331.09	440.88	440.88	440.88	440.88	440.88
28	TreC	36027.47	36027.47	36027.47	36027.47	36027.47	119049.20	119049.20	119049.20	119049.20	119049.20
29	ProC	174938.07	174938.07	174938.07	174938.07	174938.07	75917.21	75917.21	75917.21	75917.21	75917.21
30	SurC	0	0	0	0	0	0	0	0	0	0

**Table 4:** Results ($$\gamma =0.12$$)

**Scenario**		**UTC = 0.050** €$/ s \times ton$									
**USC** (€ /*ton*)		0					25				
**ECP** (€ /*ton*)		90	100	120	150	200	90	100	120	150	200
**Row**	**Label**										
1	NC	4	4	4	4	4	1	4	4	4	4
2	Nodes	1,4,9,10	1,4,9,10	1,4,9,10	1,4,9,10	1,4,9,10	10	1,4,9,10	1,4,9,10	1,4,9,10	1,4,9,10
3	Type	8,8,8,8	8,8,8,8	8,8,8,8	8,8,8,8	8,8,8,8	8	8,8,8,8	8,8,8,8	8,8,8,8	8,8,8,8
4	NP	1	1	1	1	1	1	1	1	1	1
5	Nodes	9	9	9	9	9	9	9	9	9	9
6	FW-H	1596.26	1596.26	1596.26	1596.26	1596.26	413.23	1596.26	1596.26	1596.26	1596.26
7	FW-FARM	0	0	0	0	0	0	0	0	0	0
8	FW-LP	0	0	120.38	120.38	120.38	0	0	120.38	120.38	120.38
9	FW-EC	120.38	120.38	0	0	0	1303.41	120.38	0	0	0.00
10	GW-H	691.47	691.47	691.47	691.47	691.47	214.06	691.47	691.47	691.47	691.47
11	GW-FARM	0	0	0	0	0	0	0	0	0	0
12	GW-LP	0	0	46.69	46.69	46.69	0.00	0.00	46.69	46.69	46.69
13	GW-EC	46.69	46.69	0.00	0.00	0.00	524.09	46.69	0.00	0.00	0.00
14	C-H	636.50	636.50	636.50	636.50	636.50	159.13	636.50	636.50	636.50	636.50
15	C-FARM	0	0	0	0	0	0	0	0	0	0
16	C-LP	0	0	0	0	0	0	0	0	0	0
17	C-EC	0	0	0	0	0	0	0	0	0	0
18	P-H	73.08	73.08	73.08	73.08	73.08	73.08	73.08	73.08	73.08	73.08
19	P-FARM	10.00	10.00	10.00	10.00	10.00	10.00	10.00	10.00	10.00	10.00
20	P-LP	10.00	10.00	10.00	10.00	10.00	10.00	10.00	10.00	10.00	10.00
21	P-NLP	518.01	518.01	518.01	518.01	518.01	268.94	518.01	518.01	518.01	518.01
22	SC	626.47	626.47	626.47	626.47	626.47	149.10	626.47	626.47	626.47	626.47
23	SP	0	0	0	0	0	0	0	0	0	0
24	TotC	289658.96	291329.67	293688.66	293688.66	293688.66	301234.73	306991.47	309350.46	309350.46	309350.46
25	FacC	58189.95	58189.95	58189.95	58189.95	58189.95	21606.16	58189.95	58189.95	58189.95	58189.95
26	TraC-W	7477.22	7477.22	11506.92	11506.92	11506.92	1341.70	7477.22	11506.92	11506.92	11506.92
27	TraC-P	1010.38	1010.38	1010.38	1010.38	1010.38	1159.75	1010.38	1010.38	1010.38	1010.38
28	TreC	15036.39	16707.10	15036.39	15036.39	15036.39	164475.52	16707.10	15036.39	15036.39	15036.39
29	ProC	207945.02	207945.02	207945.02	207945.02	207945.02	108924.16	207945.02	207945.02	207945.02	207945.02
30	SurC	0	0	0	0	0	3727.43	15661.80	15661.80	15661.80	15661.80

**Table 5:** Results ($$\gamma =0.12$$)

**Scenario**		**UTC = 0.150** €$/ s \times ton$									
**USC** (€ /*ton*)		0					25				
**ECP** (€ /*ton*)		90	100	120	150	200	90	100	120	150	200
**Row**	**Label**										
1	NC	3	4	5	5	6	1	3	4	5	6
2	Nodes	1,9,10	1,4,9,10	1,2,4,9,10	1,2,4,9,10	1,2,4,9,10	10	1,9,10	1,4,9,10	1,2,4,9,10	1,2,4,9,10,11
3	Type	8,8,8	7,8,8,8	8,6,7,8,8	8,6,7,8,8	8,6,7,8,8	8	8,8,8	8,7,8,8	8,6,7,8,8	8,6,7,8,8,6
4	NP	1	1	1	1	1	1	1	1	1	1
5	Nodes	9	9	9	9	9	10	9	9	9	9
6	FW-H	1191.51	1481.51	1671.70	1691.67	1716.64	413.23	1191.51	1481.51	1678.99	1716.64
7	FW-FARM	0	0	0	0	0	0	0	0	0	0
8	FW-LP	0	0	0	0	0	0	0	0	0	0
9	FW-EC	525.13	235.13	44.95	24.97	0.00	1303.41	525.13	235.13	37.65	0.00
10	GW-H	542.74	640.92	714.52	738.16	738.16	214.06	542.74	640.92	714.52	738.16
11	GW-FARM	0	0	0	0	0	0	0	0	0	0
12	GW-LP	0	0	0	0	0	0	0	0	0	0
13	GW-EC	195.41	97.23	23.63	0.00	0.00	524.09	195.41	97.23	23.63	0.00
14	C-H	477.38	588.98	664.82	677.35	684.53	159.13	477.38	588.98	666.91	684.53
15	C-FARM	0	0	0	0	0	0	0	0	0	0
16	C-LP	0	0	0	0	0	0	0	0	0	0
17	C-EC	0	0	0	0	0	1.48	0	0	0	0
18	P-H	73.08	73.08	73.08	73.08	73.08	73.08	73.08	73.08	73.08	73.08
19	P-FARM	10.00	10.00	10.00	10.00	10.00	10.00	10.00	10.00	10.00	10.00
20	P-LP	10.00	10.00	10.00	10.00	10.00	10.00	10.00	10.00	10.00	10.00
21	P-NLP	434.99	493.22	532.78	539.32	543.07	268.94	434.99	493.22	533.88	543.07
22	SC	467.35	578.95	654.79	667.32	674.50	150.58	467.35	578.95	656.89	674.50
23	SP	0	0	0	0	0	0	0	0	0	0
24	TotC	299619.72	305371.63	310665.69	312528.88	312644.54	306224.68	318508.88	326492.69	329030.17	329507.16
25	FacC	45995.36	57916.05	68795.53	68795.53	79675.01	21606.16	45995.36	57916.05	68795.53	79675.01
26	TraC-W	10739.07	13100.27	16814.43	20560.44	12022.07	4025.10	10739.07	13100.27	17358.71	12022.07
27	TraC-P	3097.79	3031.14	3007.71	3007.71	3039.07	3429.25	3097.79	3031.14	3007.71	3039.07
28	TreC	64849.44	33236.59	8229.26	3746.19	0	164475.52	72054.93	39883.91	9192.21	0.00
29	ProC	174938.07	198087.58	213818.75	216419.01	217908.38	108924.16	174938.07	198087.58	214253.84	217908.38
30	SurC	0	0	0	0	0	3764.48	11683.68	14473.74	16422.16	16862.62

**Fig. 3:**
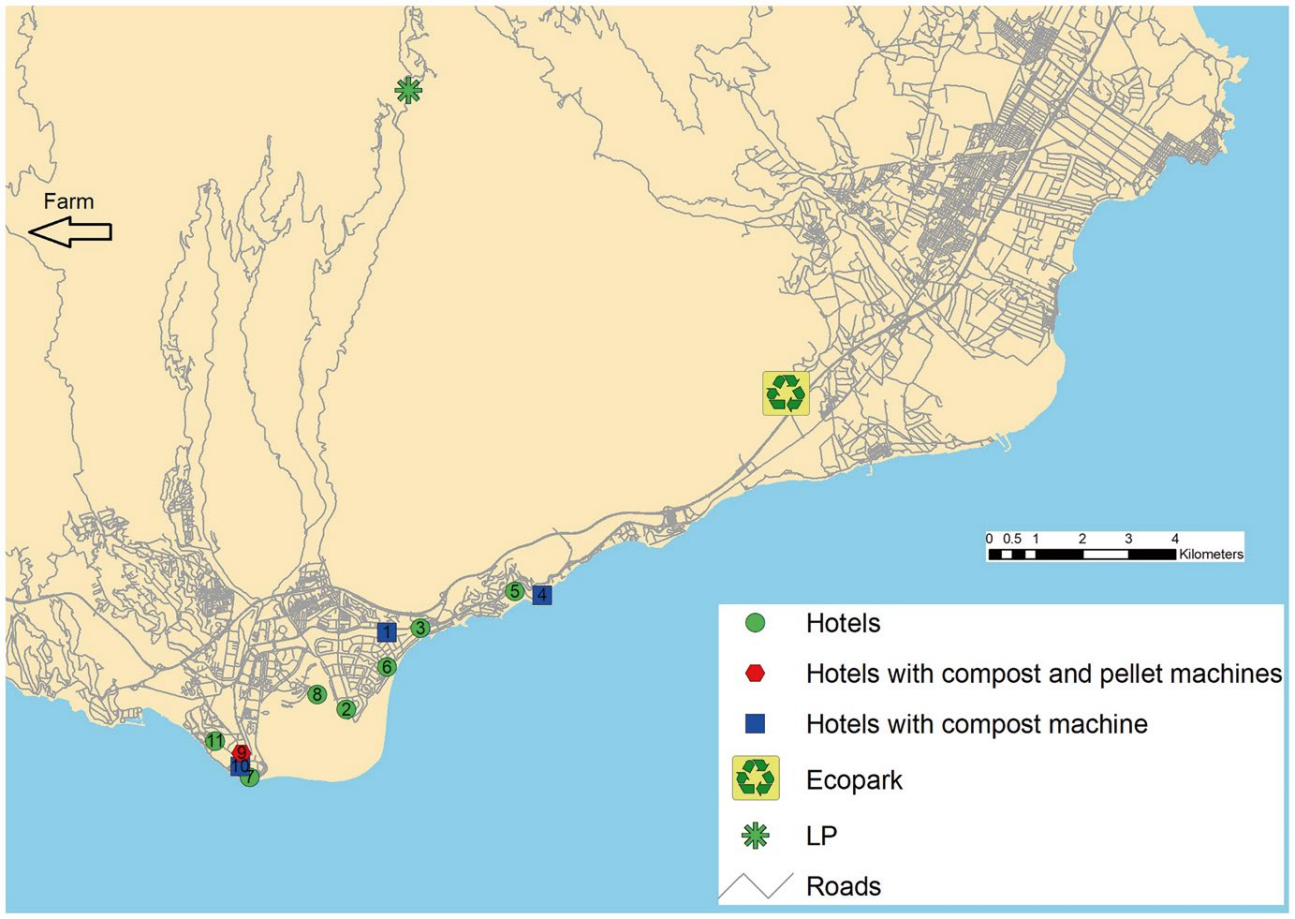
Solution for UTC = 0.05, USC = 25, and ECP = 100

**Table 6:** Facility locations for 180 scenarios

		Node						
		1	2	4	7	9	10	11
Composter								
$\gamma =0$	Type 6	0	14	0		0	0	12
	Type 7	0	9	27		0	0	0
	Type 8	54	0	25		54	55	0
	Total scenarios	54	23	52		54	55	12
$\gamma =0.05$	Type 6	0	18	0		0	0	10
	Type 7	0	0	22		0	0	0
	Type 8	53	0	27		47	50	0
	Total scenarios	53	18	49		47	50	10
$\gamma =0.12$	Type 6	0	10	0		0	0	4
	Type 7	0	0	14		0	0	0
	Type 8	40	0	21		34	43	0
	Total scenarios	40	10	35		34	43	4
Percentage (180 scenarios)		$82\%$	$28\%$	$76\%$		$75\%$	$82\%$	$14\%$
Pelletizer								
$\gamma =0$	Total scenarios	19			5	35	20	
$\gamma =0.05$	Total scenarios	19			10	29	21	
$\gamma =0.12$	Total scenarios	1			17	34	9	
Percentage (180 scenarios)		$22\%$			$18\%$	$54\%$	$28\%$	

Figure 3 shows the solution for UTC = 0.050, ECP = 100 and USC = 25, with $\gamma =0.12$. For this scenario a composter of type 8 is located at nodes 1, 4, 9 and 10, and a pelletizer is installed at node 9. We observe that for UTC = 0.050 and ECP>90, four composters are installed. Finally, Table 6 shows the number of scenarios for which a node (in column) appears in the solution as a composter and pelletizer location. Taking into account that we have 60 scenarios for each value of $\gamma$, considering the case $\gamma =0.12$, we observe that, for composters, nodes 1, 4, 9 and 10 appear in the solution for more than 50% of the cases (nodes 1 and 10 for more than 66%). Moreover, we see that the most promising node as pelletizer location is node 9, observe that nodes 7, 9 and 10 are very close, and node 1 is chosen only for one of the 60 scenarios. If we consider the 180 scenarios, the percentage of solutions in which a composter is installed at nodes 1, 2, 4, 9, 10 and 11, is $82\%$, $28\%$, $76\%$, $75\%$, $82\%$, and $14\%$, respectivelly. For pelletizers, and for nodes 1, 7, 9 and 10, this percentage is $22\%$, $18\%$, $54\%$, and $28\%$, respectively. Only composters of type 6, 7 and 8 are installed, which are the ones with the highest capacity. Moreover, type 6 appears in the solutions only for nodes 2 and 11, while type 8 appears only for nodes 1, 4, 9 and 10. Type 7 is installed only at nodes 2 and 4, much less frequently at node 2. The larger capacity composters are installed in the largest hotels, where a greater amount of waste is generated.

## Conclusions

In this paper, we present a location-allocation model to solve a bio-waste management problem in a tourism framework. The problem is to determine how many facilities to instal, where to locate the treatment facilities (composters and pelletizers) and how to allocate the bio-waste (food and garden) generated by a hotel chain and the product (compost and pellets) provided by new and existing facilities. We consider a hotel chain in the island of Gran Canaria (Spain) and build an example to illustrate the application of the mathematical programming model. Although the data used in the analysis are simulated in part, the example shows the utility of the model proposed.

From the results, and in the context of the example, we conclude that a local treatment plant that supplies pellets would satisfy part of the demand if the price were lower than or equal to the price fixed by a non-local plant. The willingness of the hotel chain to invest in treatment facilities will depend on the prices set by the existing treatment plants, the transportation costs, and the cost associated to surplus production in the hotels. Although brand image enhancement is also an incentive, we cannot forget that the hotel chain is running a business and expects its actions to contribute to increased profits. Therefore, we can suppose that innovation will be adopted faster if it is associated to cost reductions.

From the analysis of the solutions obtained for the different scenarios, we can conclude that except when the transportation and treatment cost are very low, and the surplus cost is high, the installation of composters in some hotels is profitable for the chain. In particular, nodes 1, 4, 9 and 10, are good candidates to install a composter, while node 9 is a good location for a pelletizer. We can observe that the hotels located at these nodes are the ones with the highest number of beds. Moreover, except for nodes 9 and 10, which are very close and could be considered as a unique hotel for the analysis, locations of nodes 9-10, 1 and 4 are spatially separated in the area under study.

Several extensions of the study could be tackled. From the point of view of the hotel chain, variations in the amount of waste generated, the capacity coefficient, the production coefficient, and costs, could be considered, and the implications of these changes on the solution could be analyzed. From the perspective of private investment and public managers, the possibility of increasing the local offer of bio-fuel could be studied. From the public sector perspective, policies which promote suitable environmental practices in the hospitality sector could be evaluated.

Although the illustrative example is made for a large hotel chain in an eminently tourist zone, the methodology can be extended to hotels in urban areas where guests may be travelers with reasons other than leisure, incorporating the characteristics of these hotels in the model. There are some aspects that differentiate the two contexts. On the one hand, in urban areas the concentration of large hotels of the same chain is usually less than in the case considered in Section 5, the extension of garden areas is also less, or non-existent, so the amount of garden waste may be irrelevant and the need for compost in these hotels may be less. The amount of food waste depends on the size of the hotel and the restaurant service it offers. Even considering these aspects, given that the composting process produces a significant reduction in waste, the installation of composters in this type of establishment continues to be a strategy of interest from the environmental point of view, and the compost not used in hotels can be transferred to other sectors such as agriculture, as is currently being done in some hotels. In this area, the hotel chain concept could be replaced by a network of hotels participating in the same project.

Finally, from a more global point of view, an analysis of bio-waste management on the island in the framework of a circular economy, taking into account the importance of the service sector, could provide guidelines to help achieve a more environmentally balanced waste management system in isolated territories.

## Data Availability

Data and software used are deposited in Mendeley Data: https://data.mendeley.com/datasets/c4jz5syzbp/draft?a=64fa5807-bd1e-4940-b760-90b595d3ca78
